# New insights in melanoma resistance to BRAF inhibitors: a role for microRNAs

**DOI:** 10.18632/oncotarget.26244

**Published:** 2018-10-23

**Authors:** Marta Díaz-Martínez, Lucía Benito-Jardón, Joaquin Teixidó

**Affiliations:** Marta Díaz-Martínez: Centro de Investigaciones Biológicas, Department of Molecular Biomedicine, Ramiro de Maeztu 9, Madrid, Spain

**Keywords:** miRNA, melanoma, Ras-MAPK pathway, BRAF inhibitors, resistance

The discovery of the BRAF V600E point mutation as the most frequent genetic alteration in melanoma has focused attention for the past years in the design and improvement of targeted therapy to this mutated BRAF form. Vemurafenib (VMF) and dabrafenib were generated as BRAF V600E specific inhibitors (BRAFi) providing significant therapeutic benefits for melanoma patients. However, the common emergence of drug resistance to MAPK-targeted therapy remains a challenge. Although the most frequent mechanisms involved in BRAFi resistance in melanoma converge in reactivation of the MAPK pathway, a significant portion of tumors displays resistance mechanisms that cannot be accounted for genetic alterations.

Due to the important role of microRNAs (miRNAs) in cancer progression, we recently undertook a study aimed to address the miRNA potential implication in melanoma resistance to BRAFi [1]. After generating A375 BRAF V600E melanoma cells resistant to VMF (A375-VR), we performed small RNA-seq comparing parental and VMF-resistant A375 cells. This led to the identification and validation of several miRNAs differentially expressed between both cell types, finally focusing our analyses on miR-140-3p, which was downregulated, and miR-204-5p and miR-211-5p, whose expression was increased. Changes in expression of these miRNAs in A375 cells exposed to VMF took place rapidly, were stable, and were also detected upon cell treatment with trametinib (TMT) and SCH772984, inhibitors for MEK and ERK, respectively. Furthermore, alterations in the expression of miR-204-5p, miR-211-5p and miR-140-3p were also observed in an additional BRAF V600E melanoma cell line, SK-Mel 28, and were detected in cells incubated with combined VMF and TMT, highlighting the clinical relevance of our observations. Therefore, our findings reveal close functional relationships between MAPK inhibition and changes in miR-204-5p, miR-211-5p and miR-140-3p expression, and are in line with another previous report [2]. When we looked for potential mechanisms responsible for VMF-promoted miR-204-5p and miR-211-5p upregulation, we found a role for the STAT3 transcription factor in the increased miR-204 expression, again supporting previous results [2]. Although PAX6 and MITF expression levels were enhanced by VMF, RNA silencing experiments revealed that these transcription factors did not play important roles in the increased expression of these miRNAs.

**Figure 1:**
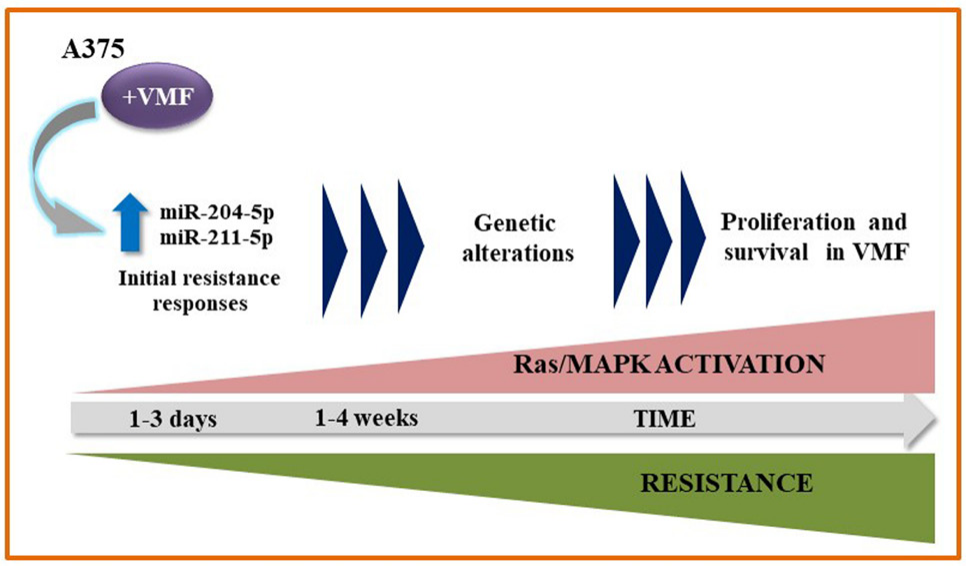
Proposed model for the contribution of miR-204 and miR-211 to VMF resistance in melanoma

Importantly, miRNA silencing and overexpression experiments demonstrated a key role for miR-204-5p and miR-211-5p, but not for miR-140-3p, in early A375 and SK-Mel28 cell resistance to VMF. Moreover, *in vivo* experiments using NSG mice revealed an increase in melanoma resistance to VMF linked to enhanced expression of these miRNAs. Of note, overexpression of miR-204 and miR-211 resulted in durable stimulation of the Ras-MAPK pathway after VMF exposure. Overall, our data strongly suggest that miR-204-5p and miR-211-5p contribute to sustained pErk1/2 levels in initials steps of melanoma resistance to VMF, allowing the later occurrence of further mechanisms to acquire full resistance (Figure 1).

Previous studies have shown the impact of miRNAs in melanoma resistance to BRAFi. Stark *et al* showed that miR-514a overexpression increased melanoma cell resistance to BRAFi, correlating with decreased expression of the tumour suppressor NF1 [3]. Another study found that miR-579-3p is down-regulated in tumour samples from melanoma patients after the development of resistance to targeted therapies. Authors demonstrated that ectopic expression of this miRNA impairs the acquisition of drug resistance in melanoma cells [4].Vergani *et al* proposed a model in which resistant cells produce increased levels of the CCL2 chemokine, which in turn alters miRNA expression, acting as facilitators of drug resistance [5]. In addition, a role for miR-7 in resistance to BRAFi has been reported. Thus, by using miR-7 mimics, it has been shown that the decreased expression of the miR-7 targets EGFR, IGF-1R and CRAF contribute to the suppression of the MAPK and PI3K/AKT pathways, reversing the melanoma cell resistance to VMF [6]. Recently, Koetz *et al* reported the upregulation of miR-125a in human melanoma cells and tissues from patients with acquired resistance to BRAFi. In this work, miR-125a was proposed to confer resistance by inhibiting pro-apoptotic components of the intrinsic apoptosis pathway [7]. Finally, a recent study addressed the RNA cargo of tumour-derived extracellular vesicles from melanoma cells after treatment with vemurafenib. It was found that this inhibitor caused changes in the RNA profile, especially an increased expression of miR-211-5p after BRAF inhibition. Furthermore, miR-211-5p transfection in melanoma cells led to increased resistance to VMF, whereas miR-211-5p inhibition in a VMF-resistant cell line reduced the cell proliferation rate [8], in close agreement with our data for the key role of this miRNA in VMF resistance.

Collectively, these results highlight the regulation by miRNAs of BRAFi resistance in melanoma, suggesting a mechanism-based strategy to limit resistance and improve clinical outcomes of melanoma patients.
